# The Association between Leucocyte Telomere Length and Survival Outcomes in Patients with Cardiovascular Disease

**DOI:** 10.31083/j.rcm2509333

**Published:** 2024-09-19

**Authors:** Jin-Yu Sun, Qian Xu, Hui Shen, Wen Huang, Qiang Qu, Wei Sun, Xiang-Qing Kong

**Affiliations:** ^1^Department of Cardiology, Gusu School, Nanjing Medical University, 215008 Suzhou, Jiangsu, China; ^2^Department of Cardiology, The First Affiliated Hospital of Nanjing Medical University, 210000 Nanjing, Jiangsu, China

**Keywords:** telomere length, cardiovascular-specific death, all-cause mortality, Fine–Gray competing risk model

## Abstract

**Background:**

We explore the association between leucocyte telomere length (LTL) and all-cause and cardiovascular disease (CVD)-specific death in CVD patients.

**Methods:**

We acquired 1599 CVD patients from a nationally representative US population survey for this study. We applied Kaplan–Meier curves, adjusted weighted Cox regression models, and restricted cubic spline to investigate the association between LTL and all-cause death. Additionally, we employed competing risk regression to assess the impact of LTL on cardiovascular-specific death, setting non-cardiovascular death as a competing event.

**Results:**

The overall mortality rate was 31.0% after a median follow-up of 13.9 years. Patients with shorter LTL exhibited a higher risk of all-cause death, with an adjusted hazard ratio (HR) of 1.25 (95% confidence interval (CI): 1.05–1.48). Restricted cubic spline illustrated a linear dose-response relationship. In gender-specific analyses, female patients with shorter LTL showed a higher risk of death (weighted HR, 1.79; 95% CI, 1.29–2.48), whereas this association was not observed in males (weighted HR, 0.90; 95% CI, 0.61–1.32). The Fine–Gray competing risk model revealed no significant relationship between LTL and cardiovascular-specific mortality but a significant association with non-cardiovascular death (adjusted HR, 1.24; 95% CI, 1.02–1.51).

**Conclusions:**

LTL is inversely associated with all-cause death in female CVD patients. The significant correlation between reduced LTL and increased all-cause mortality emphasizes LTL as a potential marker for tertiary prevention against cardiovascular disease.

## 1. Introduction

Telomeres, composed of tandem repetitive DNA sequences at eukaryotic chromosome termini, are essential for maintaining genomic stability and integrity during cell division [1, 2]. Accumulating evidence suggests that telomere lengths act as a dependable biomarker of cellular aging, serving as a mitotic clock reflecting cell division history [3, 4, 5]. Telomere length is influenced by various factors, such as age, sex, diseases, genetic variation, physiological stress, and lifestyle [6, 7]. Notably, telomere length is inversely correlated with age, and its shortening can lead to chromosomal end instability and genetic information loss [8, 9, 10].

Despite continuous efforts, cardiovascular disease (CVD) remains a major cause of mortality globally, with it responsible for 32% of deaths worldwide in 2019 [11]. Inflammation and oxidative stress have been well demonstrated as primary contributors to CVD [12, 13, 14]. Telomere shortening may exacerbate oxidative stress, leading to DNA damage, inflammation, and cellular dysfunction [15, 16, 17].

In epidemiological studies, peripheral leukocyte DNA is widely used to measure telomere length owing to the simplicity of blood sample collections [4]. Emerging research indicates that reduced leukocyte telomere length (LTL) is significantly associated with elevated mortality risk in the general population [18, 19, 20]. A study examining the general U.S. population revealed an inverse relationship between LTL and the risk of CVD [21]. Moreover, a meta-analysis has drawn attention to the link between shortened LTL and increased all-cause mortality across a broader population [22]. Recently, Xiong *et al*. [23] reported increased rates of both all-cause and CVD-related death in metabolic syndrome patients who exhibited shorter LTLs. However, the relationship between the LTL and specific outcomes for CVD patients remains uncertain. Accordingly, we explore the association between LTL and both all-cause and CVD-specific death among CVD patients in this study.

## 2. Methods

### 2.1 National Health and Nutrition Examination Survey (NHANES) and National Death Index 

The NHANES is a national cross-sectional survey evaluating the health status of U.S. individuals. The NHANES has been conducting biennial national surveys since 1999, each encompassing about 5000 nationally representative participants. The NHANES provides a comprehensive collection of demographic, physical examinations, laboratory indicators, and questionnaire information [24, 25, 26]. This study focused on only the 1999–2000 and 2001–2002 NHANES cycles, as LTL measurements were only available during these periods.

The National Death Index (NDI) database is a central repository for death records, facilitating the investigation of associations between health factors and mortality. The NDI database provides information until death or the censoring date (December 31, 2015).

**Fig. 1.**
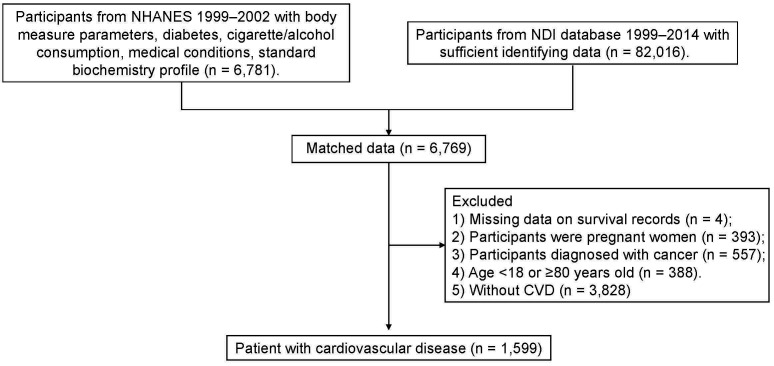
**Flow chart for participant selection**. This diagram outlines the criteria and steps to select eligible study participants from the NHANES database and the subsequent mortality tracking using the NDI. NHANES, National Health and Nutrition Examination Survey; NDI, National Death Index; CVD, cardiovascular disease.

Fig. 1 provides the flowchart for participant selection. This research included participants from the 1999–2002 NHANES cycles who had records on body measurements, diabetes, cigarette/alcohol use, medical conditions, and standard biochemistry profiles. Exclusion criteria were: (1) incomplete survival record data; (2) pregnancy; (3) cancer diagnosis; (4) age under 18 or over 79; (5) absence of CVD. Ultimately, 1599 individuals diagnosed with CVD were enrolled in the study. NHANES survey was approved by the Ethics Review Committee of the National Center, and all participants provided written informed consent.

### 2.2 Telomere Length Assessment

The methodology for telomere length measurement has been reported in previous studies [21, 27, 28]. Genomic DNA was extracted from peripheral blood samples using QIAamp 96 DNA Blood kits (Qiagen, Valencia, CA, USA). The NHANES utilized a multiplex quantitative PCR method to determine relative telomere length. This technique measures the telomere repeat copy number (T) ratio to a single-copy gene number for albumin (S), compared against a standardized reference sample. To ensure measurement consistency and accuracy, duplicate samples were assayed using the average of duplicates taken for final analysis per individual. Analytical precision was maintained by calculating mean T/S ratio values and identifying outliers by marking the highest and lowest T/S ratios. Telomere length in base pairs was converted using the following formula: kbp = 3274 + 2413 × (T/S)/1000, thereby expressing the LTL in kilobase pairs (kbp) [21, 27, 28].

### 2.3 The Definition of Cardiovascular Disease

In this research, CVD encompasses a range of conditions, including coronary heart disease, myocardial infarction, angina pectoris, heart failure, stroke, and hypertension. These diseases were identified based on participant responses to specific questions in the NHANES questionnaires. Participants were queried with the following questions: “Have you ever been told that you had a heart attack (myocardial infarction), angina pectoris, coronary heart disease, stroke, heart failure, or hypertension?” Additionally, they were asked whether they had been advised to take prescribed medication for hypertension. Affirmative responses to these questions were used as criteria to classify participants as having CVD.

### 2.4 Survival Outcomes

All-cause mortality was the primary outcome, while CVD-specific mortality was designated as the secondary outcome. Individual death statuses were obtained from the NDI database, with causes of death recorded using the International Classification of Diseases, Tenth Revision (ICD-10) [29]. Within the NDI database, the ‘MORTSTAT’ variable records the final determination of vital status, and participants coded as ‘1’ under final mortality status were considered deceased. CVD-specific death was defined as deaths resulting from heart disease (ICD codes 054-064) or cerebrovascular disease (ICD codes 070) [30]. All other causes of death were classified as non-cardiovascular.

### 2.5 Covariates

Covariates, including demographic characteristics, anthropometric measures, lifestyle factors, and medical history, were incorporated to reduce potential bias in the study [21].

Demographic information was gathered from the NHANES questionnaire, including continuous data on age and categorical data on sex (male or female), ethnicity, and educational level. Anthropometric variables included body mass index (BMI) and waist circumference. Assessed lifestyle variables included smoking status, determined by whether participants had smoked more than 100 cigarettes during their lifetime, and alcohol consumption, defined as consuming at least 12 alcoholic drinks every year. Medical comorbidities and biomarkers, such as triglycerides and diabetes, were also collected. Diabetes was defined according to the positive response to this question: “Has a doctor ever told you that you have diabetes?”

### 2.6 Statistical Analysis

We first imputed missing covariates via multivariate imputation to enhance statistical robustness [31, 32]. Normally distributed continuous variables were presented as mean ± standard deviation, while variables following a skewed distribution were shown as the median (within the quartile range), as determined by the weighted Kolmogorov–Smirnov test. Categorical variables were reported as percentages. Baseline demographic characteristics were compared using the one-way analysis of variance (ANOVA), the Kruskal–Wallis test, or the chi-square test, as appropriate.

The relationship between the LTL and overall survival was examined using the Kaplan–Meier curve for telomere length tertiles, with the Bonferroni–Holm method applied for survival comparison adjustments among groups [33, 34]. Hazard ratios (HRs) and 95% confidence intervals (CIs) were calculated by weighted Cox regression, both unadjusted and adjusted for potential confounders. LTL was both analyzed as a continuous variable and categorized into tertiles to explore its association with all-cause mortality. Model 1 was unadjusted. Model 2 was adjusted for age, sex, ethnicity, and education. Model 3 additionally considered BMI, waist circumference, triglycerides, diabetes, smoking status, and alcohol consumption alongside the Model 2 variables [30, 35]. Additionally, we utilized a weighted restricted cubic spline (RCS) with 4 knots to illustrate the correlation between LTL and all-cause death. We also performed subgroup analyses based on Cox regression, including age, gender, and diabetes.

It is important to note that Cox regression accounts for no competing risks, which could potentially lead to overestimation of absolute risks for cause-specific death. We used the Fine–Gray competitive risk regression to accurately assess the risk of LTL-related CVD-specific death, considering non-cardiovascular death as a competing outcome [36, 37]. Statistical analyses were conducted using R software (version 3.6.1, R Foundation for Statistical Computing, Vienna, Austria), with significance established at *p* < 0.05.

## 3. Results 

### 3.1 Baseline Characteristics of Participants

The average age was 59.0 years, and 50.2% of participants were males. After a median follow-up of 13.9 years, 31.0% were noted as deceased. Table 1 summarizes the demographic characteristics, anthropometric measures, behavioral factors, medical history, and plasm biomarkers of the participants, categorized by LTL tertiles.

**Table 1.** **Baseline characteristics of the study population**.

		T1 (N = 528)	T2 (N = 527)	T3 (N = 544)	*p*
Leukocyte telomere length (kbp)		5.1 (4.9, 5.3)	5.6 (5.5, 5.7)	6.2 (6.0, 6.6)	<0.001
Age (years)		65.0 (54.0, 72.0)	60.0 (47.0, 66.0)	52.0 (42.0, 63.0)	<0.001
Gender (male, n, %)		288 (54.5%)	264 (50.1%)	251 (46.1%)	0.023
Race (n, %)					<0.001
	Non-Hispanic White	277 (52.5%)	240 (45.5%)	228 (41.9%)	
	Non-Hispanic Black	116 (22.0%)	117 (22.2%)	181 (33.3%)	
	Mexican American	103 (19.5%)	123 (23.3%)	88 (16.2%)	
	Other Hispanic	21 (4.0%)	27 (5.1%)	31 (5.7%)	
	Other races	11 (2.1%)	20 (3.8%)	16 (2.9%)	
Education (n, %)					0.098
	Below high school	229 (43.4%)	213 (40.4%)	192 (35.3%)	
	High school	119 (22.5%)	120 (22.8%)	142 (26.1%)	
	Above high school	180 (34.1%)	194 (36.8%)	210 (38.6%)	
BMI (kg/m^2^)		29.5 (26.1, 34.1)	29.6 (26.4, 33.9)	29.9 (26.1, 34.5)	0.866
Waist circumference (cm)		102.8 (93.6, 114.4)	104.0 (94.5, 112.2)	102.5 (92.8, 111.6)	0.401
All-cause mortality (Yes, n, %)		234 (44.3%)	137 (26.0%)	125 (23.0%)	<0.001
Triglycerides (mg/dL)		136.5 (96.0, 191.0)	137.0 (97.0, 201.0)	128.0 (87.8, 185.0)	0.106
Diabetes (Yes, n, %)		105 (19.9%)	95 (18.0%)	94 (17.3%)	0.527
Smoking (Yes, n, %)		284 (53.8%)	286 (54.3%)	268 (49.3%)	0.193
Drinking (Yes, n, %)		99 (18.8%)	93 (17.6%)	108 (19.9%)	0.652
HF (Yes, n, %)		54 (10.2%)	33 (6.3%)	32 (5.9%)	0.011
CAD (Yes, n, %)		77 (14.6%)	43 (8.2%)	43 (7.9%)	<0.001
Stroke (Yes, n, %)		46 (8.7%)	46 (8.7%)	32 (5.9%)	0.133
Angina pectoris (Yes, n, %)		77 (14.6%)	41 (7.8%)	40 (7.4%)	<0.001
Heart attack (Yes, n, %)		89 (16.9%)	45 (8.5%)	48 (8.8%)	<0.001
Hypertension (Yes, n, %)		463 (87.7%)	486 (92.2%)	515 (94.7%)	<0.001

T1, 4.21–5.37 kbp; T2, 5.37–5.85 kbp; T3, 5.85–8.98 kbp. BMI, body mass index; HF, heart failure; CAD, coronary artery disease.

There is a significant difference across the tertiles (T1–T3) in age, race, gender, and prevalence of CVD (*p* < 0.05). No significant disparities were noted in BMI (*p* = 0.866) and waist circumference (*p* = 0.401).

### 3.2 The Association of LTL with All-Cause Mortality 

**Fig. 2.**
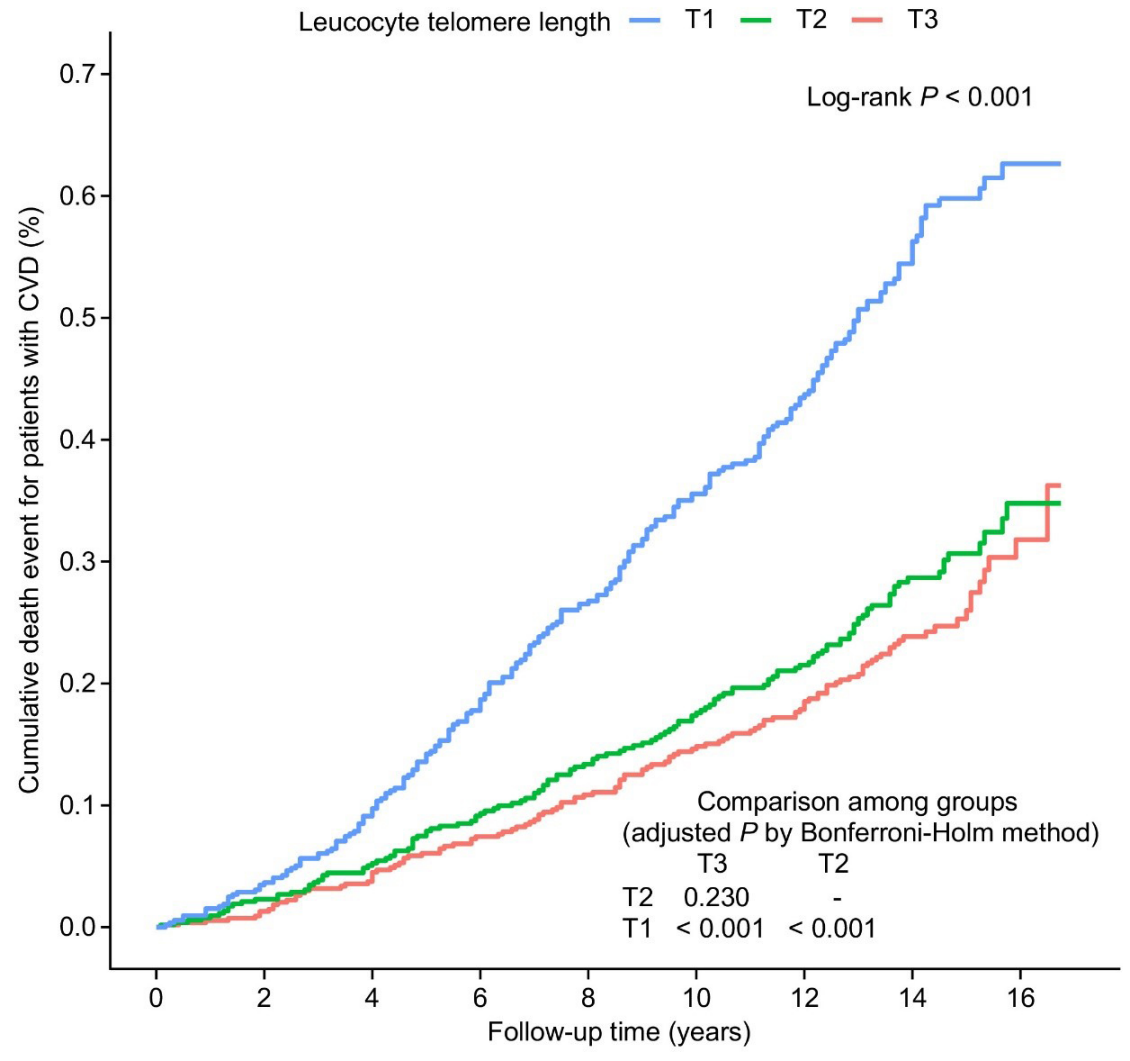
**Kaplan–Meier curves by LTL tertiles in patients with CVD**. The LTL tertiles, based on the entire CVD patient cohort, are T1 (<5.37 kbp), T2 (5.37–5.85 kbp), and T3 (>5.85 kbp). LTL, leucocyte telomere length; CVD, cardiovascular disease.

Kaplan–Meier curve analysis revealed significant differences in all-cause mortality among the LTL tertiles (log-rank *p* < 0.001). As shown in Fig. 2, individuals with lower LTL had substantially reduced survival rates compared to those with higher LTL.

LTL significantly correlates with all-cause mortality across all models (Table 2). Specifically, HRs with 95% CIs were 2.00 (1.68–2.37) for Model 1, 1.27 (1.07–1.51) for Model 2, and 1.25 (1.05–1.48) for Model 3. In the categorical analysis (non-adjusted, Model 2, and Model 3), an increased risk of all-cause death was observed with decreasing LTL categories. In Model 3, participants in the lowest LTL tertile (T1) showed a 1.29-fold increased risk of death than the highest tertile (T3). However, we observed no significant difference in death risk between the middle tertile (T2) and the highest tertile (T3) (*p* = 0.196).

**Table 2.** **The association between leucocyte telomere length and all-cause mortality based on Cox regression**.

		Model 1		Model 2		Model 3	
		HR (95% CI)	*p*	HR (95% CI)	*p*	HR (95% CI)	*p*
LTL (per 1 kbp decrease)		2.00 (1.68, 2.37)	<0.001	1.27 (1.07, 1.51)	0.007	1.25 (1.05, 1.48)	0.011
Categories							
	T3 (>5.85 kbp)	Reference		Reference		Reference	
	T2 (5.37–5.85 kbp)	1.16 (0.91, 1.48)	0.232	0.87 (0.68, 1.11)	0.259	0.85 (0.66, 1.09)	0.196
	T1 (<5.37 kbp)	2.24 (1.80, 2.78)	<0.001	1.30 (1.04, 1.63)	0.023	1.29 (1.03, 1.62)	0.028

HR, hazard ratio; LTL, leucocyte telomere length; CI, confidence interval; T1, tertile 1; T2, tertile 2; T3, tertile 3.

The dose-response association of LTL with all-cause death was further explored using RCSs, with the median LTL serving as the reference point. As illustrated in **Supplementary Fig. 1**, all-cause death was elevated with decreasing LTLs after adjusting for covariates such as age, sex, race, and educational attainment.

**Fig. 3.**
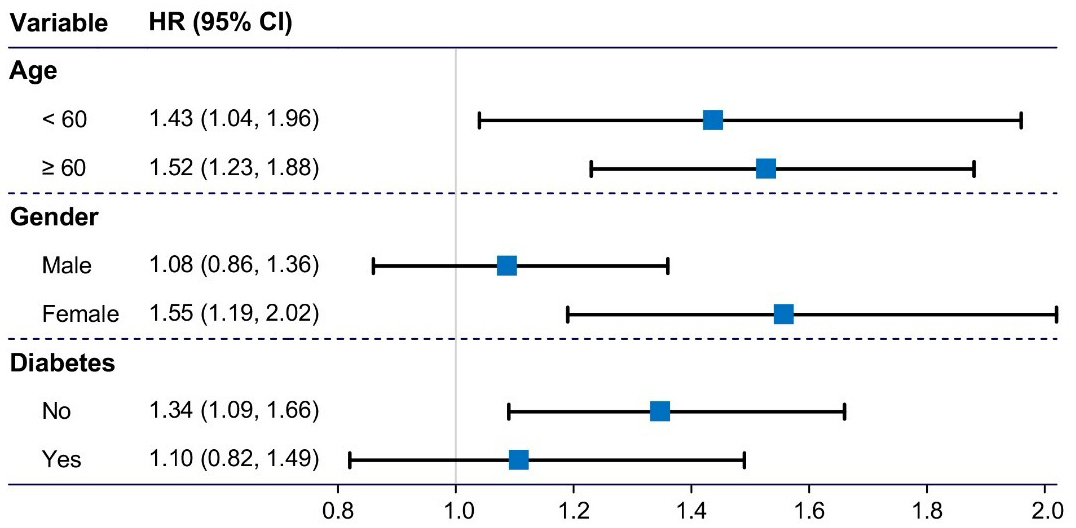
**Subgroup analysis on the associations of LTL with all-cause death across age, gender, or diabetes**. The associations were adjusted for age, race, education, body mass index, waist circumference, triglycerides, diabetes, and smoking and drinking statuses. The related covariate was removed from the model when the group was divided based on this covariate. HR, hazard ratio; CI, confidence interval; LTL, leucocyte telomere length.

As shown in Fig. 3, the association between LTL and all-cause death remained robust regardless of age (<60 or ≥60 years). Interestingly, the association was not significant in the male group, whereas in the female subgroup, the HR was more pronounced at 1.55 (95% CI, 1.19–2.02). Table 3 illustrates the weighted association between LTL and all-cause death by gender. When LTL was analyzed as continuous, it showed a significant association with increased all-cause mortality in the female group (weighted HR, 1.79; 95% CI, 1.29–2.48), although this association was not observed in the male group. In the categorical analysis, females exhibited a higher all-cause death risk with decreasing LTL categories, highlighting a gender-specific disparity in the impact of telomere length on mortality risk.

**Table 3.** **The weighted association between LTL and all-cause death in different genders**.

			HR (95% CI)	*p*
Male				
	LTL (per 1 kbp decrease)		0.90 (0.61, 1.32)	0.591
	Categories			
		T3 (>5.85 kbp)	Reference	
		T2 (5.37–5.85 kbp)	0.64 (0.40, 1.00)	0.051
		T1 (<5.37 kbp)	0.94 (0.66, 1.34)	0.722
Female				
	LTL (per 1 kbp decrease)		1.79 (1.29, 2.48)	<0.001
	Categories			
		T3 (>5.85 kbp)	Reference	
		T2 (5.37–5.85 kbp)	0.78 (0.57, 1.07)	0.917
		T1 (<5.37 kbp)	1.28 (0.94, 1.74)	0.004

We adjusted for age, race/ethnicity, education level, body mass index, waist circumference, triglycerides, diabetes, and smoking and drinking statuses. Abbreviations: HR, hazard ratio; CI, confidence interval; LTL, leucocyte telomere length.

### 3.3 The Association between LTL and CVD-specific and non-CVD-specific Death

**Fig. 4.**
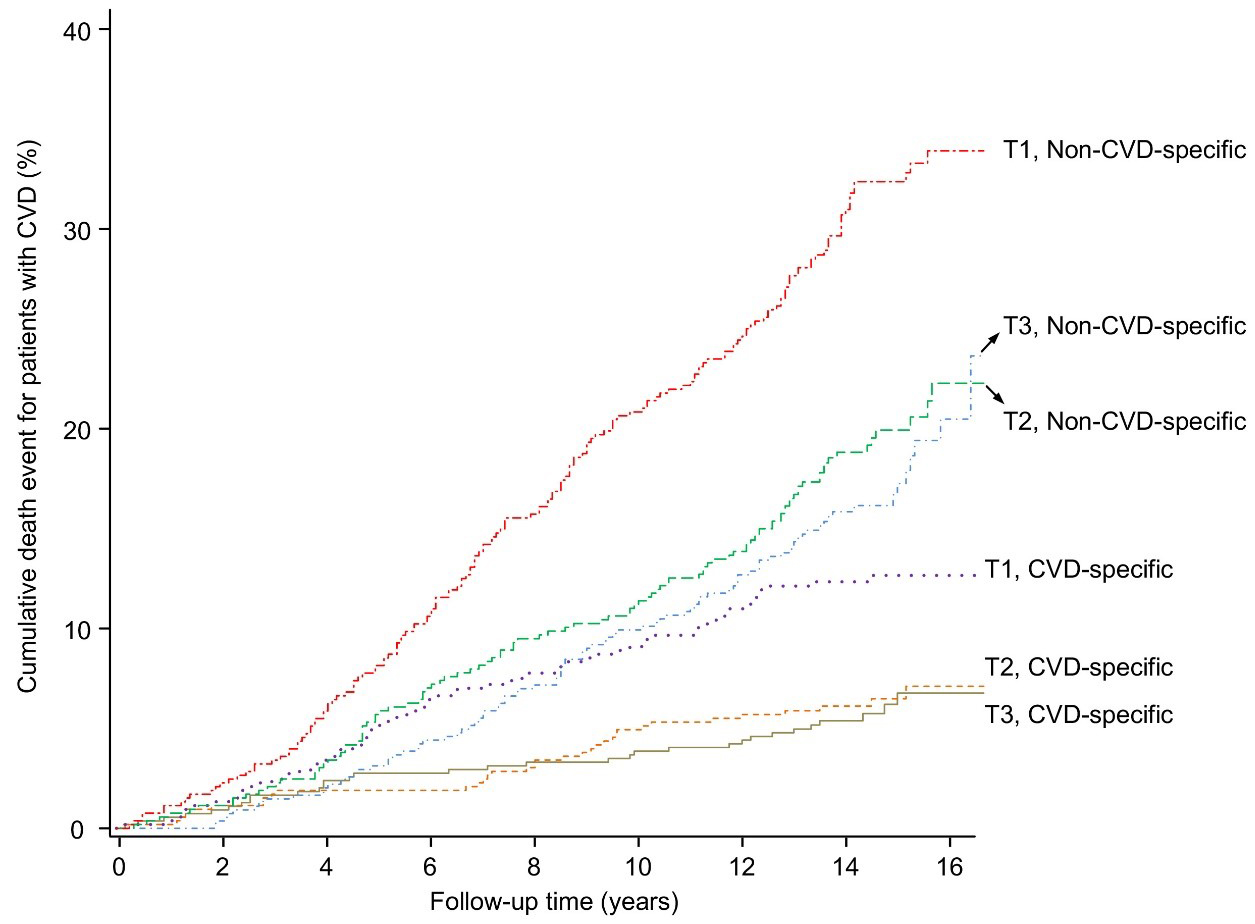
**Cumulative incidence of cause-specific death in LTL tertiles**. Cumulative incidence of both CVD-specific and non-CVD-specific mortality across different LTL tertiles in CVD patients. The LTL tertiles, based on the entire CVD patient cohort, are T1 (<5.37 kbp), T2 (5.37–5.85 kbp), and T3 (>5.85 kbp). LTL, leucocyte telomere length; CVD, cardiovascular disease.

The cumulative incidence of cause-specific death in the LTL tertiles is shown in Fig. 4.

After integrating LTL into the Fine–Gray competing risk model, we initially observed a significant association between LTL and both cardiovascular and non-cardiovascular mortality. Specifically, for cardiovascular death, the HR was 2.01 with a 95% CI of 1.37–2.93; for non-cardiovascular death, the HR was 1.84 (95% CI, 1.51–2.24), without adjusting for covariates.

However, after adjusting for covariates, the association between LTL and CVD-specific mortality was no longer significant (adjusted HR, 1.21; 95% CI, 0.85–1.74). In contrast, the association between LTL and non-CVD death remained statistically significant even after adjustments, with an adjusted HR of 1.24 (95% CI, 1.02–1.51).

## 4. Discussion

Telomeres shorten with each cell division, eventually becoming critically short and fragile after numerous divisions [3, 38]. LTL serves as a biomarker, indicative of the overall telomere length in an organism. CVD encompasses a spectrum of heart and blood vessel disorders, including hypertension, atherosclerosis, myocardial infarction, and so forth [11], and it remains a major cause of mortality from non-communicable diseases [39].

In our investigation, which included 1599 CVD patients from the 1999 to 2002 NHANES, we examined the relationship between LTL and the prognosis of CVD. Notably, individuals in the low LTL group displayed an increased all-cause death than those in the high LTL group. Kaplan–Meier curve analyses revealed a marked difference in all-cause mortality across LTL tertiles (log-rank *p* < 0.001). To delve deeper into the impact of LTL on all-cause mortality, we employed adjusted Cox regression to assess the association of LTL (as a continuous and categorical variable) with the risk of all-cause mortality. As a continuous variable, LTL was associated with an adjusted HR of 1.25 (95% CI, 1.05–1.48). The lowest LTL group (T1) had a significantly higher risk compared to the highest group (T3), with HR values of 1.29 (95% CI, 1.03–1.62). The RCS analysis indicated a linear dose-response relationship between LTL and all-cause mortality, with increased mortality risk accompanying decreasing LTL. While LTL showed no significant association with CVD-specific death in the competing risk model, it remained significantly associated with non-CVD death (adjusted HR: 1.24; 95% CI, 1.02–1.51).

Numerous studies have examined the length of telomere and survival outcomes [19, 20, 40]. A recent meta-analysis involving 121,749 individuals found a short LTL correlated with increased all-cause mortality in the general population [22]. Schneider *et al*. [10] observed a similar association between shortened LTL and all-cause, circulatory, and respiratory mortality based on 472,432 participants from UK Biobank. Recently, Xiong *et al*. [23] observed increased all-cause and CVD-specific mortality in patients with metabolic syndrome who had shorter LTL. However, the specific association between LTL and mortality in patients with CVD needs to be clarified.

Our study corroborates these findings and extends them to individuals with CVD, demonstrating that shorter LTL is associated with higher all-cause mortality, even considering multiple demographic and clinical factors. The long follow-up of our study highlights the important role of LTL as a prognostic biomarker in clinical practice, particularly for identifying high-risk groups among CVD patients.

Interestingly, our findings regarding CVD-specific mortality align with several previous studies [41, 42, 43] in demonstrating no significant association between LTL and cardiovascular mortality risk. Mons *et al*. [41] reported no correlation between telomere length and CVD-specific death in 12,199 participants from two population-based prospective cohort studies. In contrast, Xiong *et al*. [23] revealed that LTL was independently associated with CVD-specific mortality. This discrepancy between studies may stem from several factors. It should be noted that oxidative stress plays a critical role in telomere shortening and potentially impacts our findings. Oxidative stress leads to oxidative DNA damage, including telomere attrition, which is a key factor in aging and the pathogenesis of various diseases, including CVD [14, 44]. However, our research adjusted for multiple covariates that influence oxidative stress (e.g., age, sex, race, BMI, and lifestyle), although other potential covariates remain to be considered. Second, there is a discrepancy in the research population. Xiong *et al*. [23] focused on individuals with metabolic syndrome, whereas our study population comprised patients with CVD. Third, previous analysis employed the Cox regression model to examine cause-specific mortality, which may not adequately account for biases introduced by competing outcomes, potentially leading to overestimating the risk of CVD-specific death [23]. Our study utilized the competing risk regression, which sets non-cardiovascular death as a competing event, potentially providing a more accurate assessment of cardiovascular mortality risk. These findings indicate the complexity of telomere biology and its implications for disease. Therefore, additional research is necessary to clarify the relationship between LTL and cause-specific mortality. This could involve more extensive studies with diverse populations and methodologies, ensuring a comprehensive understanding of how LTL influences mortality risks in various contexts of cardiovascular health.

Moreover, our research found that LTL is inversely related to all-cause death in female patients with CVD, which was not observed in males. Males and females often experience varying aging rates, leading to differences in life expectancy between genders [45]. Females possess, on average, longer telomeres and a higher life expectancy than males of equivalent age [9, 45]. In humans, males live shorter lives and experience more rapid telomere shortening, leading to speculation in medical research that gender-specific telomere reduction might be a contributing factor to the differences in gender-specific death [45]. However, Xiong *et al*. [23] did not observe consistent results, and gender variables in subgroup analyses did not significantly alter the association between telomere length and risk of all-cause or CVD-specific death. Apart from the difference in the study population, their neglect of weighted study design is another possible cause, potentially leading to a conclusion containing selection bias.

Despite the novel perspectives on LTL and the prognosis of CVD, the limitations of this study should be mentioned. Firstly, while comprehensive, focusing on a nationally representative sample from the United States does not account for variations in physical activity, dietary, genetic factors, and other potential covariates. Moreover, the generalizability of our conclusion to populations in different regions, such as China and Europe, still needs to be determined due to the lack of representative data from these areas. These limitations suggest that more research should be performed to validate and expand our findings, ideally incorporating more diverse and global populations and a broader range of covariates.

## 5. Conclusions

Our study demonstrates that telomere length is inversely related to all-cause mortality in individuals with CVD. This finding reveals that shorter LTL correlates with an elevated mortality risk from all causes. However, this relationship does not relate to CVD-specific mortality. The significant link between reduced LTL and all-cause death underlines the importance of considering telomere length as a biomarker in managing CVD. By identifying patients with shorter LTLs, healthcare providers can potentially implement more targeted interventions and monitoring strategies, aiming to improve overall outcomes and reduce mortality risks in individuals with CVD.

## Availability of Data and Materials

All the data used in this study were acquired from the NHANES survey (https://wwwn.cdc.gov/nchs/nhanes/Default.aspx).

## Supplementary Material

Supplementary material associated with this article can be found, in the online version, at https://doi.org/10.31083/j.rcm2509333.
