# Supplementary material for: The Association between Leucocyte Telomere Length and Survival Outcomes in Patients with Cardiovascular Disease
Source: Rev Cardiovasc Med. 2024 Sep 19;25(9):333. doi: 10.31083/j.rcm2509333 (PMC11440408; doi:10.31083/j.rcm2509333)
Supplement: Supplementary file 1 [file 2153-8174-25-9-333-s1.docx]

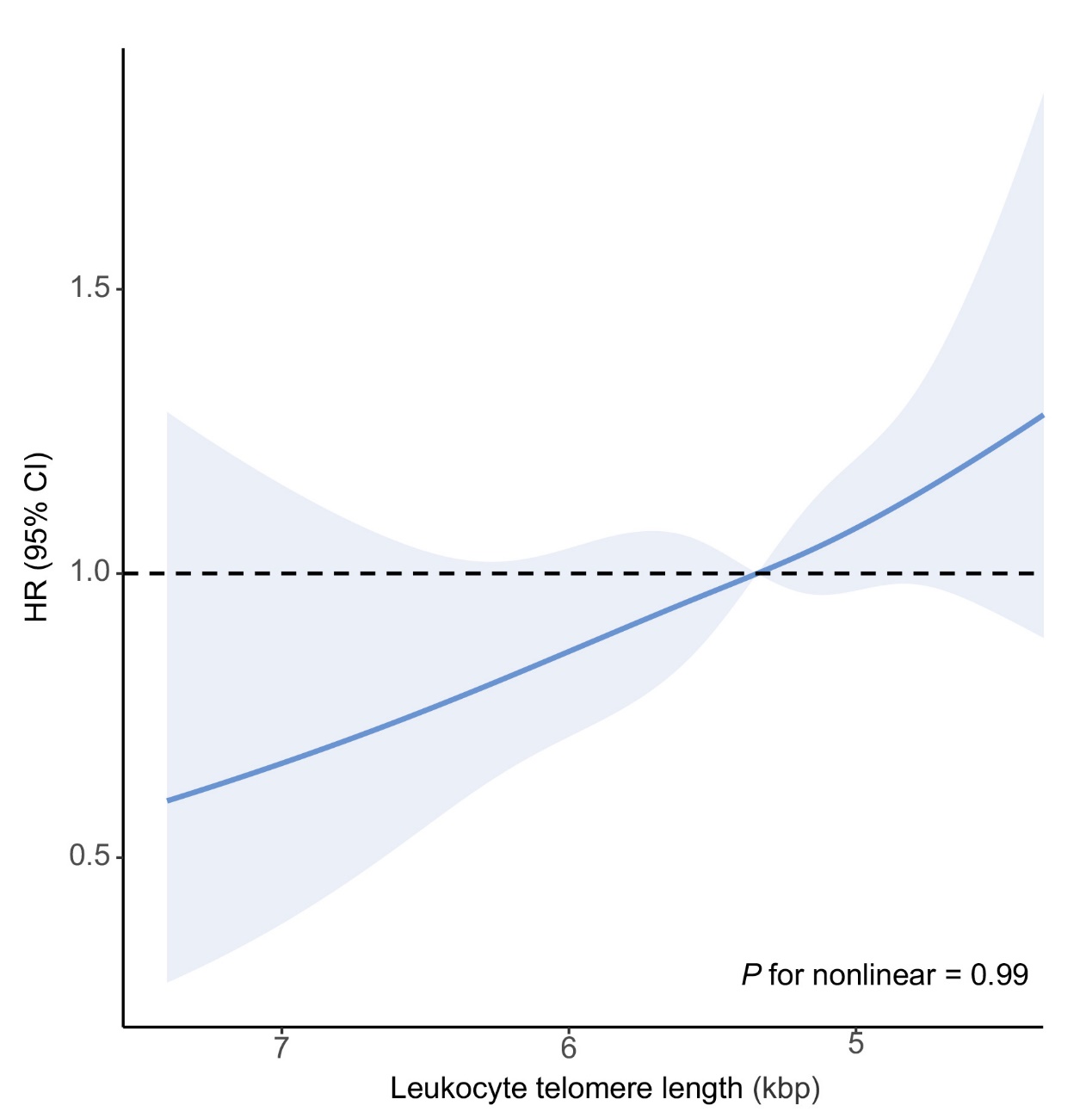


**Supplementary Fig. 1.** **Cox regression-based restricted cubic spline on the association between LTL and all-cause death.** Covariates were adjusted for, including age, race, gender, and education. LTL: leucocyte telomere length.
